# Internalized FGF-2-Loaded Nanoparticles Increase Nuclear ERK1/2 Content and Result in Lung Cancer Cell Death

**DOI:** 10.3390/nano10040612

**Published:** 2020-03-27

**Authors:** Tianxin Miao, Andrew C. Little, Alexander Aronshtam, Taylor Marquis, Spencer L. Fenn, Milena Hristova, Dimitry N. Krementsov, Albert van der Vliet, Jeffrey L. Spees, Rachael A. Oldinski

**Affiliations:** 1 Bioengineering Program, College of Engineering and Mathematical Sciences, Larner College of Medicine, College of Engineering and Mathematical Sciences, University of Vermont, Burlington, VT 05405, USAspencer.fenn@uvm.edu (S.L.F.); 2 Cellular, Molecular and Biomedical Sciences Graduate Program, University of Vermont, Burlington, VT 05405, USA; andrew.little@uvm.edu (A.C.L.); albert.van-der-vliet@med.uvm.edu (A.v.d.V.); 3 Department of Pathology and Laboratory Medicine, Larner College of Medicine, University of Vermont, Burlington, VT 05405, USA; milena_big@yahoo.com; 4 Department of Medicine, Stem Cell Core, Larner College of Medicine, University of Vermont, Colchester, VT 05446, USA; Alexander.Aronshtam@uvm.edu (A.A.); taylor.marquis@uvm.edu (T.M.); 5 Department of Biomedical and Health Sciences, College of Nursing and Health Sciences, University of Vermont, Burlington, VT 05405, USA; Dimitry.Krementsov@uvm.edu; 6 Department of Mechanical Engineering, College of Engineering and Mathematical Sciences, University of Vermont, Burlington, VT 05405, USA; 7 Department of Electrical and Biomedical Engineering, College of Engineering and Mathematical Sciences, University of Vermont, Burlington, VT 05405, USA; 8 Materials Science Program, College of Engineering and Mathematical Sciences, University of Vermont, Burlington, VT 05405, USA

**Keywords:** nanoparticles, lung cancer, intracellular FGF-2, cancer cell death, ERK1/2

## Abstract

Innovative cancer treatments, which improve adjuvant therapy and reduce adverse events, are desperately needed. Nanoparticles provide controlled intracellular biomolecule delivery in the absence of activating external cell surface receptors. Prior reports suggest that intracrine signaling, following overexpression of basic fibroblast growth factor (FGF-2) after viral transduction, has a toxic effect on diseased cells. Herein, the research goals were to (1) encapsulate recombinant FGF-2 within stable, alginate-based nanoparticles (ABNs) for non-specific cellular uptake, and (2) determine the effects of ABN-mediated intracellular delivery of FGF-2 on cancer cell proliferation/survival. In culture, human alveolar adenocarcinoma basal epithelial cell line (A549s) and immortalized human bronchial epithelial cell line (HBE1s) internalized ABNs through non-selective endocytosis. Compared to A549s exposed to empty (i.e., blank) ABNs, the intracellular delivery of FGF-2 via ABNs significantly increased the levels of lactate dehydrogenase, indicating that FGF-2-ABN treatment decreased the transformed cell integrity. Noticeably, the nontransformed cells were not significantly affected by FGF-2-loaded ABN treatment. Furthermore, FGF-2-loaded ABNs significantly increased nuclear levels of activated-extracellular signal-regulated kinase ½ (ERK1/2) in A549s but had no significant effect on HBE1 nuclear ERK1/2 expression. Our novel intracellular delivery method of FGF-2 via nanoparticles resulted in increased cancer cell death via increased nuclear ERK1/2 activation.

## 1. Introduction

Lung cancer is one of the most prevalent types of carcinoma, resulting in the largest number of cancer-related deaths worldwide [1,2,3]. Greater than 85% of lung cancer cases are classified as non-small-cell lung cancer (NSCLC), including adenocarcinoma, squamous-cell carcinoma, and large-cell carcinoma [4]. Despite recent advances in early detection and cancer treatment, NSCLC is often diagnosed at a late stage in the disease and bears poor prognosis [1] Standard of care treatment for NSCLC includes surgical resection of pulmonary lesions, combined with radiotherapy [5] or chemotherapy to prevent or reduce tumor-induced symptoms [6]. Unfortunately, chemotherapy is curative in only a small proportion of patients and is frequently associated with systemic toxicity [7,8] and long-term adverse events (AEs) [9]. Accordingly, new therapeutic strategies with improved fatal targeting of cancer cells and reduced off-target toxicity are widely sought after to provide stand-alone cancer treatments or adjunct treatments that allow for reduced use of chemotherapeutic(s).

Paracrine and autocrine basic fibroblast growth factor (FGF-2) signaling contributes significantly to NSCLC invasion and survival [10,11,12]. FGF-2 is a secreted, extracellular ligand that binds, with varying degrees of affinity, all four-cell surface FGF receptors (FGFR1-4). Upon cell surface binding, FGF-2/FGFR interaction promotes receptor dimerization, trans-phosphorylation of tyrosine kinase domains [13,14], and commonly results in increased cancer cell survival, proliferation, drug resistance, and neoangiogenesis [15]. FGFR2 stimulates the Rat sarcoma/mitogen-activated protein kinase (Ras/MAPK) signaling pathway consisting of ERK1/2 [10,11,12]. The ERK1/2 pathway is a master regulator of cell fate decisions in eukaryotes (i.e., cell proliferation, survival, and transformation, or cell death [16,17,18]. ERK1/2-mediated phosphorylation of FGFR2 constitutes a negative feedback loop that regulates FGFR2 expression. In cancer cells, this pathway is broken, and FGFR2 is overexpressed [19]. Inhibition of the FGFR mediated ERK1/2 pathway, using a MEK1/2 inhibitor, increased FGFR2 signaling. Indeed, ERK1/2 activation does not require FGFR activity; the level and location of ERK1/2 activation are important to investigate. The distribution of ERK1/2 is governed by the stimulus concentration, the route of stimulation (i.e., location), and the duration of stimulation. While FGFR inhibitors and FGF traps have been proposed and tested, systemic AEs arise [20,21,22]. Alternatively, intracellular FGF activity has been investigated as an important target for cancer therapy.

High (23 kDa) and low molecular weight (18 kDa) FGF2 isoforms can have similar, as well as isoform-specific, effects on cell proliferation, differentiation, migration, and survival, and accumulate in varying proportions in different cell types [23,24]. In nontransformed cells, 18 kDa FGF-2 is often found, whereas the 23 kDa FGF2 is more often present inside of transformed cells, including breast cancer cells (MCF-7). Therefore, a difference exists in the transformed cells regarding the FGF-2 isoform that is present. Interestingly, increasing either the cytosol levels of 18 kDa or 23 kDa FGF-2 reduced cell proliferation by inhibiting cell-cycle progression and protein translation [25]. Published data also show that increasing intracellular FGF-2 of a pancreatic cell line modulated protein kinase C delta to activate ERK1/2 [23].

The difference in ERK1/2 signaling leading to cell proliferation or programmed death lies in the cellular location in which ERK1/2 is activated [16,17,18,26]. Nuclear activation of ERK1/2 upregulates p53, a pro-apoptotic protein known to induce chromatin compaction and cell death [27,28]. Signal transduction through ERK/p53 has been shown to induce cell apoptosis after various chemotherapeutic treatments [27,28,29,30], whereas loss of p53 was shown to promote cell proliferation by activating a Raf/Mek/ERK signaling pathway [31]. In contrast to nuclear activation of ERK1/2, phosphorylated ERK1/2, located in the cell cytoplasm, can inhibit nuclear ERK1/2 and induce cell proliferation [29]. Recent studies indicate that elevated levels of cytosolic low molecular weight FGF-2 (18 kDa) successfully activated intracellular ERK1/2, which reduced breast cancer cell (MCF-7) migration and proliferation [32] The recombinant FGF-2 (both 18 kDa and 23 kDa) intracellular delivery inhibited MCF-7 proliferation and induced downregulation of B-cell lymphoma 2 (Bcl-2) and increased levels of Bax, without involving receptor-mediated signaling [32]. Bax promotes apoptosis, while Bcl-2 is a survival factor. In light of the observed differences in the pattern of FGF-2 localization, the administration of agents capable of blocking both the paracrine and intracrine activities of FGF-2 in NSCLC cells has been suggested as a potential approach to cancer treatment [33].

Particle encapsulation is a core technology used in drug delivery systems [34] and encompasses a wide range of polymeric materials. Drug micro-encapsulation provides the following benefits: (1) uniform particle size and shape; (2) a protective barrier from the extracellular environment; (3) controlled temporal and spatial drug release [35]. Alginate, a derivative of brown seaweed, is readily formed into microparticles using ionic crosslinking and used to deliver proteins, cytokines, cells, and various small molecules [36,37,38]. However, non-modified microparticles produced with traditional fabrication techniques often exhibit relatively large diameters, which hinder cell internalization and the controlled rate of drug-release [39]. Furthermore, the anionic properties of natural polymers, such as alginate, can impede cellular uptake. On a smaller scale, nanoparticles encompass an assortment of colloidal nano-systems (≤100 nm) capable of passing through biological barriers (i.e., passive targeting), and encapsulating drugs at effective concentrations. In this manner, administered nanoparticles may enhance chemotherapeutic efficacy through intracellular delivery relative to approaches that employ systemic or exogenous drug delivery [40,41].

To facilitate the intracellular delivery of FGF-2 and determine its effects on NSCLC cell proliferation, survival, and intracrine signaling, we synthesized a neutralized alginate-graft-poly(ethylene glycol) (Alg-*g*-PEG) copolymer and ionically crosslinked it to form Alg-*g*-PEG-based nanoparticles (ABNs). Here we report on ABN characterization and drug loading and the routes of cellular uptake, internalization, and ERK1/2 pathway for a tumorigenic, malignant cell line and an immortal, but non-tumor forming cell line treated with control ABNs and FGF-2-loaded ABNs. Our results demonstrate differential effects of internalized FGF-2-loaded ABNs on the cytosolic and nuclear ERK1/2 pools that correlate with decreased viability, survival, and proliferation of tumorigenic cells, but not of non-tumorigenic immortalized cells.

## 2. Materials and Methods

### 2.1. Materials

Sodium alginate (M_w_ = 65–75 kg/mol, 60–70% guluronic acid residues) was generously donated by FMC BioPolymer (Philadelphia, PA, USA). Amine-poly(ethylene glycol)-thiol (NH_2_-PEG-SH, M_w_ = 1000 g/mol) and methyl-poly(ethylene glycol)-amine (mPEG-NH_2_, M_w_ = 500 g/mol) were purchased from Laysan Bio (Arab, AL, USA). N-ethyl-N′(3-dimethylaminopropyl) carbodiimide hydrochloric acid (EDC), N-hydroxysuccinimide (NHS), 2,2′-dithiodipyridine, methanol (MeOH, anhydrous, 99.8%), biology-grade mineral oil, Span 80, 3-(4,5-dimethylthiazol-2-yl)-2,5-diphenyltetrazolium bromide (MTT)-based In Vitro Toxicology Assay Kit, cholera toxin, bovine serum albumin (BSA), and dexamethasone were purchased from Sigma-Aldrich (St. Louis, MO, USA). One molar hydrochloric acid (HCl) and 1 M sodium hydroxide were purchased from VWR (Philadelphia, PA, USA). Sodium citrate, isopropanol, calcium chloride (CaCl_2_), sodium chloride (NaCl), magnesium chloride (MgCl_2_), Alexa Fluor^®^ 647 Cadaverine, 20x phosphate buffered saline (PBS), 4% paraformaldehyde (PFA) in PBS, Triton^®^ X-100, 4-(2-hydroxyethyl)-1-piperazineethanesulfonic acid (HEPES), glycerol, phenylmethylsulfonyl fluoride (PMSF), sodium orthovanadate (Na_3_VO_4_), aprotinin, leupeptin, a bicinchoninic acid assay (BCA) kit, a subcellular protein fractionation kit for cultured cells, BupH™ MES Buffered Saline Packs, Roswell Park Memorial Institute (RPMI) 1640 Medium, and a Pierce LDH Cytotoxicity Assay Kit were purchased from Thermo-Fisher Scientific (Waltham, MA, USA). Fetal bovine serum (FBS) was purchased from Atlanta Biologicals (Flowery Branch, GA, USA). Dulbecco’s Modification of Eagle’s Medium (DMEM, glutaGRO™)/F-12 media, penicillin, streptomycin, and trypsin EDTA were purchased from Corning Cellgro (Oneonta, NY, USA). A human FGF enzyme-linked immunosorbent assay (ELISA) Kit was purchased from BioLegend (San Diego, CA, USA). Human anti-FGF-2 antibody and immunoglobulin G (IgG) (rabbit) were purchased from Abcam (Cambridge, MA, USA). Human epidermal growth factor (EGF), insulin, transferrin, and bovine pituitary extract (BPE) were purchased from Lonza (Portsmouth, NH, USA). Adenosine triphosphate (ATP)-based Cell Titer-Glo^®^ assay reagent was purchased from Promega (Madison, WI, USA).

### 2.2. Cloning, Expression, and Purification of Basic Human FGF (FGF-2)

FGF-2 coding sequence from a human cDNA library was PCR-modified by adding a Kpn1 restriction site to the 5′-end and an Xho1 restriction site to 3′-end. Following digestion, it was cloned into pCR4.TOPO (Invitrogen); this vector served as a shuttle vector. For protein expression, we cloned the insert into plasmid pET32b (Novagen®); this vector helps to prevent oxidation of labile cysteine disulfide groups by generating a fusion between the protein of interest (i.e., FGF-2) and thioredoxin. To produce the FGF-2-thioredoxin fusion protein, the expression plasmid was transformed into *E. coli* strain BL21 (DE3) [42]. Bacteria were grown in Luria–Bertani (LB) broth with ampicillin and vigorous shaking and induced by IPTG (0.2 mM) at 25 °C for 4–5 h. Bacteria were pelleted by centrifugation (16,000× *g* for 5 min), re-suspended into sodium phosphate buffer with 5 mM imidazole (0.05 M NaPO_4_, 0.2 M NaCl, pH 7.5) and then disintegrated by sonication. Cell extract was spun down (36,000× *g* for 10 min), and the supernatant was applied onto 1 mL of Ni-NTA resin (ThermoFisher). The FGF-2-thioredoxin fusion protein was eluted with phosphate buffer containing 400 mM imidazole. Protein concentration was monitored by Bradford reaction using a microplate format. Protein composition and yield of FGF-2-thioredoxin fusion protein in supernatant was verified by SDS-NuPAGE™ minigels (Novex) stained with Coomassie Brilliant Blue R. Eluate from the Ni-NTA column was desalted (Sephadex G-25, 20 mL column) and treated with thrombin (0.01 μM) (Haematologic Technologies, Essex Junction, VT, USA) overnight at ambient temperature to cleave thioredoxin from the fusion protein. The digested mixture was applied to a 1 mL heparin-sepharose column (GE Healthcare) that was pre-equilibrated with PBS. Thioredoxin was exclusively found in the flow-through, whereas FGF-2 was retained on the column. Purified FGF-2 was eluted from the heparin-sepharose column with PBS containing 1.5 M NaCl.

### 2.3. FGF-2-Loaded ABNs

ABN fabrication was based on our previous work, including Alg-*g*-PEG synthesis [41,43]. FGF-2 was added to a 1% (w/v) Alg-*g*-PEG solution at a weight ratio of 1:10^5^ (FGF-2:polymer). ABNs without FGF-2 were fabricated as blank controls. At room temperature, 1 mL of Alg-*g*-PEG/FGF-2 solution was added to 6.72 mL of 5% (v/v) Span 80 in mineral oil and mixed at 1200 rpm for 5 min. Next, 400 µL of 30% (v/v) Tween 80 in mineral oil was added, followed by the slow addition of 5 mL of 2 M CaCl_2_ solution. The emulsion was mixed for 30 min, 3 mL of isopropanol was added, and then the emulsion was centrifuged at 4000 rpm for 5 min. ABNs were washed sequentially with isopropanol (×2) and DI water (×2). The hydrodynamic diameter and zeta-potential of ABNs were determined using dynamic light scattering (DLS, Zetasizer Nano ZSP, Malvern, Malvern, United Kingdom) in PBS at pH = 7.4, 37 °C. ABNs were flash-frozen in liquid N_2_, lyophilized, and characterized by scanning electronic microscopy (SEM, JEOL 600, JEOL, Peabody, MA, USA); samples were sputter-coated with 45 nm of gold/palladium.

### 2.4. FGF-2 Encapsulation Efficiency and Release

Lyophilized FGF-2-loaded ABNs were dissolved in 3% (w/v) sodium citrate solution [44]. The FGF-2 concentration was measured using a BioLegend ELISA Development Kit according to the manufacturer’s protocol. FGF-2 concentration was determined against a standard curve, and the FGF-2 encapsulation efficiency (mass of FGF-2 encapsulated in the ABNs / mass of FGF-2 added when forming the ABNs) was calculated.

### 2.5. Cellular Uptake of ABNs

A549 adenocarcinoma cells were obtained from the American Type Culture Collection (ATCC, CCL-185^TM^, Gaithersburg, MD, USA). HBE1 cells were generated by Yankaskas et al. (1993) through the infection of immortalized human bronchial epithelial cells with papillomavirus [45]. Both cell types were seeded separately into 6-well plates at 3 × 10^5^ per well and cultured until they reached 80% confluency. A549 culture medium contained DMEM, 10% FBS, and 1% penicillin/streptomycin. HBE1 culture medium contained DMEM, cholera toxin (10 ng/mL), EGF (10 ng/mL), transferrin (5 μg/mL), bovine pituitary extract (15 μg/mL), and BSA (0.5 mg/mL). ABNs were labeled with AlexaFluor 647 via carbodiimide chemistry and added to cells at 100 µg/mL (n = 3). After 12 h of culture, the medium was removed, and adherent cells were thoroughly rinsed with PBS. Cells were trypsinized and centrifuged at 200× *g* for 10 min, and then re-suspended and fixed in 1 mL of 4% PFA in PBS for 10 min. After fixation, cells were centrifuged to remove excess PFA and thoroughly rinsed with 1× PBS. Cells were re-suspended in sterile PBS and transferred to 5 mL polystyrene round-bottom tubes for flow cytometry to determine the percentage of the cell population that internalized ABNs (BD LSRII Flow Cytometer, San Jose, CA, USA). Alexa 647-positive cell population percentages were gated with non-treated cells and those treated with non-labeled ABNs.

### 2.6. Route of Internalization and Intracellular Localization

Blank ABNs were labeled with AlexaFluor 647 via carbodiimide chemistry, and suspended in medium with various blockers of endocytosis: (1) chlorpromazine hydrochloride (CH) to inhibit clathrin-mediated endocytosis [46] (10 mg/mL); (2) nystin (NY) to inhibit caveolar-mediated endocytosis [47] (25 µg/mL); (3) colchicine (CO) to inhibit micropinocytosis [48] (40 µg/mL); and (4) dynasore (DY) to inhibit dynamin (80 µM) [49,50,51]. A549s were seeded in 6-well plates at 3 × 10^5^ per well and cultured until they reached 80% confluency. Cells were incubated in the presence of blank AlexaFluor 647-labeled ABNs (n = 3) at 100 µg/mL, 37 °C and 5% CO_2_. After 30 min, the culture medium was removed, and adherent cells were thoroughly rinsed with sterile PBS to remove non-internalized ABNs. Cell samples were prepared for flow cytometry (vide supra). A Tukey statistical test was performed to compare the difference of the percentage of cells with ABNs between non-blocked groups, and blocked groups. To verify that fluorescent signals were originating from internalized ABNs and not membrane-bound ABNs, the same cell samples were characterized using confocal laser scanning microscopy (CLSM, Zeiss LSM 510 META, White Plains, NY, USA). Z-stack images were obtained with AimImage Software. For MTT-based cytotoxicity assays, A549 and HBE1 cells were also prepared for MTT-based cytotoxicity assays. Adherent cells were thoroughly rinsed with PBS, and the mitochondrial activity was determined using an MTT-based assay, per the manufacturer’s protocol. Experimental sample absorbance values were normalized to cell only controls to calculate the percentage of mitochondrial activity for each treatment type. Data are represented as mean ± standard deviation (n = 3; replicated 2×).

To track ABN internalization, A549s were incubated with AlexaFluor 647-labeled ABNs (100 µg/mL) and rhodamine-labeled dextran (12.5 mg/mL) for 10 and 30 min, and 3 and 24 h [52,53]. Cells with non-labeled dextran, and without any treatments, were prepared as controls. At different time points, the medium was removed, and adherent cells were rinsed thoroughly with PBS. Cells were prepared for CLSM (*vide supra*) and z-stack images were obtained.

A549s were seeded on sterile Nunc™ Thermanox™ Coverslips in 6-well plates overnight in preparation for transmission electron microscopy (TEM). ABNs (100 µg/mL), were added to the medium and co-cultured for 10 and 30 min, and 1, 4, 8, 12, 24, and 48 h. At each time point, the medium was removed, and cells were thoroughly rinsed with PBS. Cells were fixed for 30 min in Karnovsky’s fixative (2.5% glutaraldehyde, 1% PFA in 0.1 M PBS) at 4 °C, rinsed with Millonig’s phosphate buffer, and post-fixed in 1% osmium tetroxide (OsO4) in 0.1 M cacodylate buffer at 4 °C for 30 min. Samples were dehydrated in a graded series of ethanol, through propylene oxide, and infiltrated and embedded in Spurr’s resin. Ultra-thin sections were cut with a diamond knife, placed onto 200 mesh nickel thin-bar grids, and contrasted with alcoholic uranyl acetate and lead citrate. Grids were viewed with a TEM (JEOL 1400 USA, JEOL, Peabody, MA, USA) operating at 60 or 80 kV, and digital images were acquired with an AMT-XR611 11 megapixel CCD camera [54,55].

### 2.7. Cell Toxicity Assays

For cell viability and LDH-based cytotoxicity assays, cells were seeded at 1 × 10^4^ per well into the black, flat-bottomed 96-well plates for top-down fluorescence readings. Cells were cultured in RPMI 1640 (no FBS) in the presence of the various treatments (mentioned previously), for 24 h. Post 24 h, medium containing various components was aspirated, and cells were provided fresh full growth medium for an additional 24 h, after which cell viability was determined using ATP TiterGlo assay reagent per manufacturer’s instructions. LDH release (measure of cell integrity) assays were performed in an identical fashion per manufacturer’s directions (Pierce LDH Cytotoxicity Assay Kit). Data are represented as average ± standard deviation (* *p* < 0.05; ** *p* < 0.01 were calculated by one-way ANOVA, n = 6, replicated 2×).

To control for potential differences resulting from the growth media for A549 and HBE1 cells, we also performed assays using a mixed A549/HBE1 cell growth media (1:1). For these experiments, each cell type was initially grown in its respective medium and then expanded for two days in mixed media. At 80% confluency, cells were washed once in PBS and then incubated for 24 h with ABNs (with or without FGF-2) resuspended in the mixed A549/HBE1 cell media (1:1). Cell numbers were quantified with the use of a dye-binding assay to detect nucleic acid (CyQuant Direct Assay, n = 3, repeated 3×). To determine whether extracellular FGF-2 could alter the cellular response to ABN exposure, we compared the number of A549s and HBE1s that were incubated for 24 h with FGF-2 loaded ABNs to those that were exposed to extracellular FGF-2 (20 ng/mL) coincident with FGF-2 loaded ABN treatment. As above, these experiments were carried out using mixed A549/HBE1 media (1:1).

### 2.8. Western Blot

The production of ERK1/2 proteins in A549 and HBE1 cells, after treatment with ABNs, was determined by immunoblotting. Four control and experimental groups were prepared: (1) cells without any treatment; (2) cells with FGF-2-loaded ABNs; (3) cells with extracellular (free) FGF-2 (20 ng/mL); (4) cells with blank ABNs. Cells were plated and cultured until 80% confluent, and the different treatments were added to the cultures for 24 h. For whole-cell protein analysis, cells were lysed using 200 μL of 1× western solubilization lysis buffer (1% Triton X-100, 50 mM HEPES, 250 mM NaCl, 10% glycerol, 1.5 mM MgCl, 1 mM PMSF, 1 mM EDTA, 2 mM Na_3_VO_4_, 10 μg/mL aprotinin, 10 μg/mL leupeptin), pH 7.4, per well. For cellular fraction protein analysis, protein samples from membrane/cytosol and nuclei fractions were obtained using a subcellular protein fractionation kit based on the manufacturer’s protocols. Equal amounts of protein (20–25 μg; determined using BCA protein assay) were separated on Novex 10% Tris-Glycine gels, transferred to nitrocellulose membranes and blotted using antibodies against pERK-1/2 (#4370; 1:1000; Cell Signaling) and ERK1/2 (#4695; 1:1000; Cell Signaling) [51,56]. Quantified western blot data are representative of two separate experiments.

## 3. Results and Discussion

### 3.1. ABN Fabrication and Characterization

**Figure 1:**
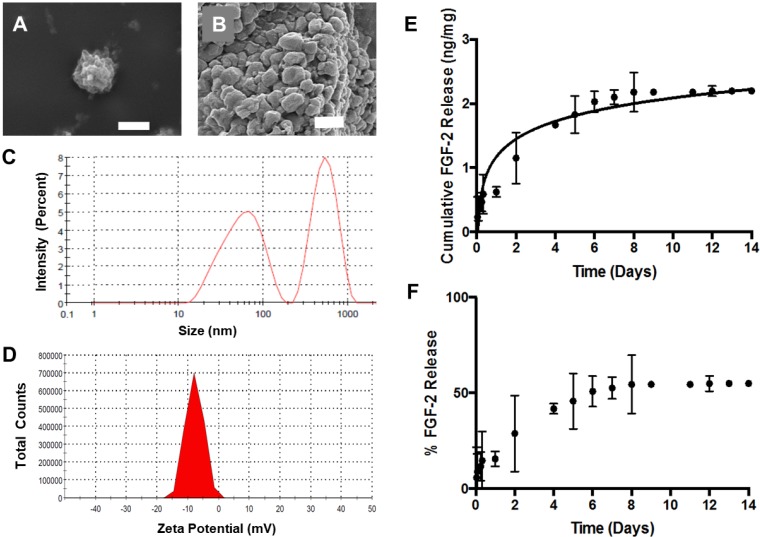
Scanning electron micrographs depicting spherical lyophilized alginate-based nanoparticles (ABNs): (**A**) scale bar = 1 µm, and (**B**) scale bar = 500 nm. (**C**) Hydrodynamic diameter distribution of fibroblast growth factor 2 (FGF-2)-loaded ABNs, measured using dynamic light scattering (DLS) in the buffered saline, pH 7.4, 37 °C, and reported as intensity-average mean; (**D**) hydrodynamic zeta-potential distribution measured in buffered saline, pH 7.4, 37 °C, and reported as number-average mean. (**E**) The cumulative mass of FGF-2 release from 5 mg ABNs was calculated over 14 days (100 µg/mL ABNs) in the buffer, pH 7.4 and 37 °C; FGF-2 loading (ng FGF-2/mg ABN) was measured by testing solution aliquots using an enzyme-linked immunosorbent assay (ELISA). (**F**) Approximately 50% of the loaded FGF-2 was released from ABNs after 14 days.

Blank and FGF-2-loaded ABNs were fabricated using a water/oil emulsion based on our previous reports [41,57]. Scanning electron micrographs of lyophilized ABNs depict a textured yet uniform surface, and a relatively uniform, spherical morphology (Figure 1A,B). While the ABNs appear heterogeneous in size (Figure 1B), the range is small, even in the dehydrated state. DLS further analyzed the size and surface charge (i.e., zeta-potential) of hydrated ABNs in the hydrated state. The greatest population of particles, as measured by DLS, had average diameters by intensity and volume of 521 nm and 170 nm, respectively (Figure 1C). The ABNs exhibited an increased surface charge -8 mV (Figure 1D). The electronegativity was drastically reduced after a theoretical 10% PEG copolymerization compared to nonmodified alginate nanoparticles; PEG copolymerization reduces interference with the negative cell membrane [38,43]. As indicated by the ABN characterization, the physical properties were optimal for cell internalization.

For our experiments, we cloned, expressed, and purified recombinant human FGF-2 from *Escherichia coli* (*E. coli*), using a two-step affinity chromatography purification procedure (Supplementary Figure S1). The encapsulation efficiency of FGF-2 (i.e., the amount of protein retained during ABN fabrication) was 80%. Release of FGF-2 from ABNs in buffered saline at 37 °C was quantified over a period of two weeks using an ELISA. The FGF-2 diffusion-based release occurred up to eight days, beyond which subsequent release was negligible; an approximate concentration of 2 ng FGF-2 released per 1 mg ABN was maintained (Figure 1E). The data confirm that FGF-2 was loaded into ABNs via encapsulation, and approximately 85% of the total encapsulated FGF-2 was retained within the ABNs post-one-day release (Figure 1F), which is a considerably high drug content retention well within the window of ABN uptake. Thus, while some FGF-2 was released from the ABNs, a majority of FGF-2 was encapsulated in the ABNs during the time frame of in vitro cell culture. In addition, the influence of free FGF-2 was investigated as a control in a later experiment (*vide infra*).

### 3.2. ABN Internalization and Endocytosis Assays

**Figure 2:**
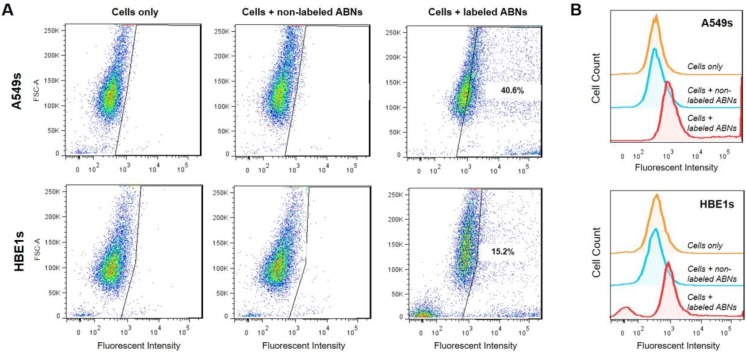
(**A**) Flow cytometry was used to determine A549 (top panel) and HBE1 (bottom panel) cancer and nontransformed ABN positive cell populations after 12 h of culture with no treatment, treatment with non-labeled ABNs, or treatment with AlexaFluor 647-labeled ABNs (100 µg ABNs/mL media). (**B**) Cell count curves plotted on a log scale for control and experimental groups.

Non-treated cells, A549s, and HBE1s treated with non-labeled blank ABNs, or AlexaFluor 647-labeled ABNs, were cultured for 12 h to allow for cellular ABN uptake; the ABN concentration in the culture media was 100 mg/mL. Flow cytometry demonstrated that >40% of A549s and >15% of HBE1s internalized AlexaFluor 647-labeled ABNs (Figure 2A). Histograms reveal clear shifts in mean fluorescent intensity (MFI) between control and experimental groups for each cell type, demonstrating successful uptake of blank ABNs by both A549s and HBE1s. MFI values for non-treated cells and for cells treated with non-labeled ABNs were relatively close, whereas the MFI values for AlexaFluor 647-labeled ABN-treated cells more than doubled compared to the controls (Figure 2B). Flow cytometry yields semi-quantitative results for fluorescence either taken up by cells or attached to their outer membrane but cannot distinguish between attachment and intracellular uptake. Thus, further characterization was utilized to confirm the internalization of ABNs within transformed cells, the cell target of interest.

**Figure 3:**
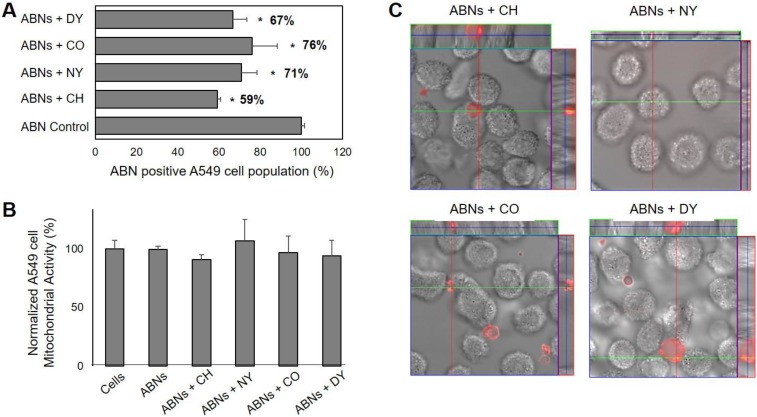
(**A**) Endocytosis-dependent A549 cancer cell uptake of ABNs suspended in cell media (100 µg/mL). Results are presented as a percentage of the ABN-positive A549 cell population after treatment with various endocytosis blockers (chlorpromazine hydrochloride (CH), nystin (NY), colchicine (CO), dynasore (DY)); ANOVA, * p < 0.01 versus ABN control, n = 3. Clathrin-inhibitors resulted in the greatest reduction in uptake for ABN-positive cell populations. (**B**) The combined effect of ABN exposure and inhibition of endocytosis on percent cellular mitochondrial activity in A549s after 30 min of culture was not significant from the control group. (**C**) Confocal laser scanning z-stack merged micrographs confirmed AlexaFluor 647-labeled ABNs were located inside the cytoplasm of A549s.

We analyzed specific endocytic pathways, which may be used by A549s to internalize ABNs, using blockers to inhibit endocytosis. Indeed, compared with the control group (without any blockers), all four blockers significantly decreased ABN uptake, suggesting that a combination of multiple pathways may be involved, or uptake is non-specific [58,59]. The inhibition of macropinocytosis resulted in the smallest change, reducing ABN uptake by 24%. Inhibition of clathrin-mediated endocytosis decreased ABN uptake by 41% (Figure 3A), suggesting that clathrin-mediated endocytosis played a key role in ABN uptake. Indeed, these results align with published results that particles <200 nm in size are internalized via clathrin-mediated pits, correlating with ABN dimensions supported by the DLS measurements [60]. We also verified that the cellular mitochondrial activity of A549s was not significantly affected by the inhibition of endocytosis (Figure 3B), demonstrating the reduction in ABN uptake was not correlated with impaired cell viability. To confirm that ABNs were indeed internalized, CLSM was performed. The examination of the z-stack merged images confirmed that AlexaFluor 647-labeled ABNs were located inside the cytoplasm of A549s (Figure 3C).

### 3.3. Intracellular Localization of ABNs

**Figure 4:**
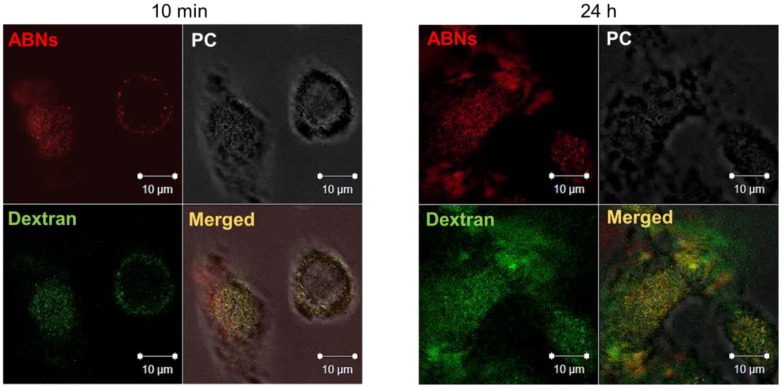
Confocal laser scanning z-stack merged micrographs of A549 cancer cells cultured with AlexaFluor 647-labeled ABNs at 100 µg/mL (red) and rhodamine-labeled dextran (green) after 10 min (left panel) and 24 h of incubation (right panel). PC = phase contrast.

ABN cellular uptake and endocytic vesicle-mediated intracellular localization of AlexaFluor 647-labeled ABNs was confirmed by culturing A549s with rhodamine-labeled dextran, typically taken up by endocytosis and enclosed in membrane-bound vesicles during intracellular transport (e.g., endosomes and lysosomes). After 10 min of culture, fluorescent signals for both ABNs and dextran were visible and merged CLSM images verified co-localization of ABNs within dextran-labeled vesicles (Figure 4). The CLSM data agreed with flow cytometry results and verified that ABNs were internalized within membrane-bound vesicles. After 24 h of culture, there appeared to be less overlay between dextran-labeled lysosomes and ABNs, suggesting that ABNs were escaping lysosomes or ABNs were being broken down (Figure 4). We hypothesize that the mechanism of FGF-2 escape may occur during ABN swelling within the relatively low-pH of lysosomes, thereby causing ABN porosity to increase, releasing FGF-2 within the cytoplasm. Indeed, future work can examine FGF-2 intracellular trafficking [61,62].

**Figure 5:**
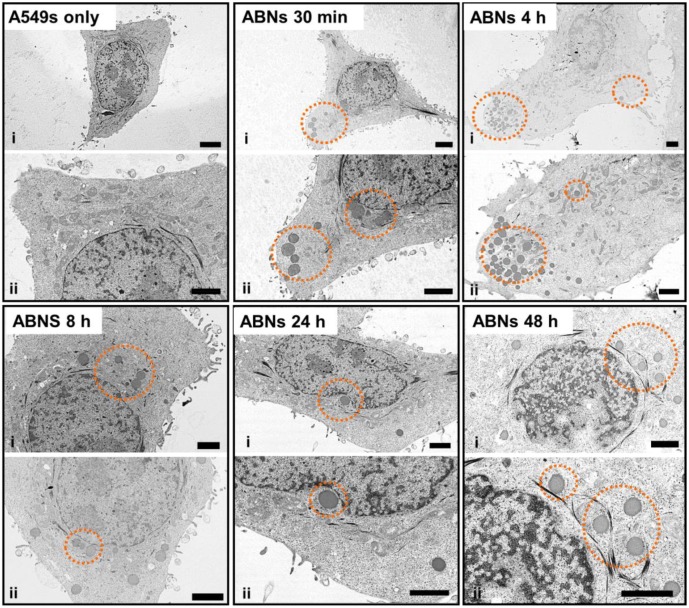
TEM images of A549 cancer cells without exposure to blank ABNs (A549s only) or after incubation with blank ABNs (100 µg/mL) for 30 min to 48 h. Two images at different magnifications are shown for each time point (scale bar = 2 µM). Dashed orange circles indicate blank ABNs within A549s.

Compared with images of non-treated (control) A549s, TEM images of treated A549s demonstrated that ABNs were distinguishable from intracellular compartments and organelles due to their dark contrast and spherical shape (Figure 5). In the TEM images, the extension of the cell membrane and endocytosis were seen 30 min post-incubation. At 4 h post-incubation, higher concentrations of ABNs were seen within the cells, close to the cell membrane, and ABNs located at the edge of the cell membrane were still detectable. At 8 h post-incubation, ABN density decreased in the cytoplasm; however, ABNs within the cytoplasm localized close to the nucleus. At 24- and 48-h post-incubation, ABNs localized to the nucleus and appeared to interact with the nuclear membrane.

### 3.4. Bioactivity of FGF-2-Loaded ABNs

**Figure 6:**
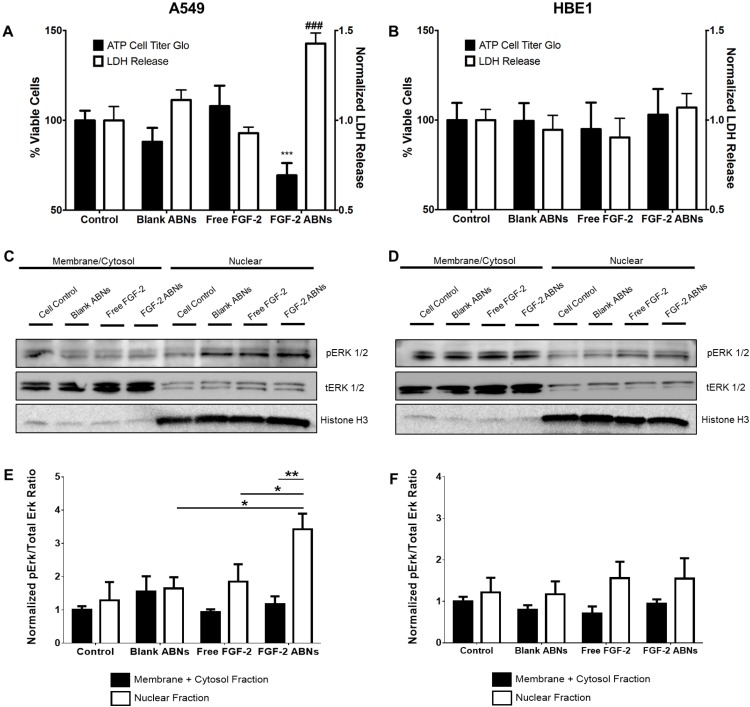
In vitro normalized viable cell number and LDH release for (**A**) A549 cancer and (**B**) HBE1 nontransformed cells after exposure to blank ABNs (100 µg/mL), free FGF-2 (20 ng/mL), and FGF-2-loaded ABNs (100 µg/mL). Western blot gel band images of membrane/cytosol and nuclear pERK1/2 and tERK1/2 activation for (**C**) A549 and (**D**) HBE1 cells. Relative intensity ratios of western blot gel bands for pERK/tERK ratios in both membrane/cytosol and nuclear fractions for (**E**) A549 and (**F**) HBE1 cells.

ABN and FGF-2 bioactivity were evaluated to determine the effectiveness of our intracellular delivery system. As expected, due to innocuous properties of the alginate base material, blank ABNs had no significant effect on cell viability, or cell integrity, on either cell type. After 24 h of co-culture with FGF-2-loaded ABNs, A549 viability significantly decreased compared with blank ABNs and non-treated cell controls (Figure 6A). A549 release of lactate dehydrogenase (LDH) was significantly increased after FGF-2-loaded ABN treatment, again showing the significant effect of intracellular FGF-2-loaded ABNs to decrease cancer cell line integrity. Notably, no significant effects on cell viability or LDH release were observed for HBE1s after exposure to FGF-2 loaded ABNs (Figure 6B), indicating that the toxic effects of FGF-2-loaded ABNs may be selective to tumorigenic cells.

Western blots were performed to determine whether the cytotoxic effects of FGF-2-loaded ABNs correlated with changes in ERK1/2 activation, indicating a similar result with increased FGF-2 cytosol content in cancer cell lines. Whole cellular protein lysates were examined, and no significant difference was observed between treatment groups for either cell type (Supplementary Figure S2). Next, we quantified ERK1/2 activation in different cellular fractions (membrane/cytosol and nucleus). Immunoblotted protein bands for phosphorylated (activated) ERK (pERK1/2) and total ERK (tERK1/2) are shown in Figure 6C,D. Nuclear ERK1/2 activation in A549s treated with FGF-2-loaded ABNs was significantly higher compared to the control and other treatment groups (Figure 6C). The amount of activated nuclear ERK1/2 associated with exogenous FGF-2 was not significantly different from the control. Most notably, the level of nuclear ERK1/2 activation in HBE1s that possess a more differentiated phenotype relative to A549s was not affected by FGF-2-loaded ABN treatment (Figure 6D). Thus, we found that the distribution and quantity of ERK1/2 were both affected by the route of FGF-2 administration (extracellular or intracellular) and cell type.

To control for potential differences resulting from the growth media for A549 and HBE1 cells, we also performed assays using a mixed A549/HBE1 cell growth medium (1:1). From the experiments performed, it could not be determined if the premature release of FGF-2 from the ABNs influenced cell viability, so further supplementation of the medium was done. Empty ABNs and FGF-2-loaded ABNs in the presence of exogenous FGF-2 were compared to the cell viability of FGF-2-loaded ABNs with cell medium containing no exogenous FGF-2. The simultaneous incubation of A549 cells with extracellular FGF-2 (free ligand) and FGF-2 loaded ABNs did not alter the ability of FGF-2 ABNs to reduce A549 cell numbers relative to control A549 cells that were treated with empty ABNs alone (Supplementary Figure S3).

While the focus for this study was to determine if ABNs can transport FGF-2 into a cell to induce a toxic response, the study only analyzed ERK1/2 activation and no other signaling pathways. As shown in the literature, increasing the level of intracellular FGF-2 by viral transduction was reported to activate death-associated protein kinase to promote nuclear transport of ERK1/2 and apoptosis [63]. Furthermore, increasing the cytosol level of FGF-2 reduced glioma cell proliferation in vivo by inhibiting cell-cycle progression and protein translation [25]. Studying a pancreatic cell line (AR42J), Gaubert et al. demonstrated that intracellular FGF-2 modulated protein kinase C delta (PKC-δ) to activate ERK1/2 [23,46]. In addition to the cytoplasm, FGF-2 has also been shown to localize to the cell nucleus [64,65]. Claus et al. demonstrated that FGF-2 contained a C-terminal NLS that directed it more specifically to Cajal bodies and the nucleolus [65]. In our work, nuclear pERK1/2 levels in A549s, a cancer cell line, correlated with increased LDH release. On the contrary, HBE1 cells exhibited lower levels of nuclear pERK1/2 and no significant changes in cell viability. Of special interest, the differences we observed between A549s and HBE1s may relate to the relative degree of transformation; however, this was not the focus of this study and was not investigated further. Alternatively, the intracellular (cytoplasmic) release of FGF-2 from the ABNs may have negatively altered A549 viability in a different manner. For example, activation of ERK1/2 might occur indirectly, because of jeopardized and dying cells. Additional studies are warranted to determine whether FGF-2-loaded ABN uptake by cancer cells influences FGFR dynamics or intracrine signaling pathways to decrease NSCLC survival.

## 4. Conclusions

This study is the first to report on the intracellular delivery of FGF-2 by ABNs and its cytotoxic effects on a cancer cell line. We demonstrated successful cellular uptake of ABNs by flow cytometry, fluorescence microscopy, and electron microscopy, and our data suggest ABNs are internalized through non-specific endocytosis. For cancer cells, intracellular delivery of FGF-2 via ABN uptake produced cytotoxic effects that correlated with significantly higher levels of activated nuclear ERK1/2. In contrast, the transformed cells, which are immortal but non-tumorigenic, did not respond significantly when treated with FGF-2-loaded ABNs. In addition, the presence of exogenous FGF-2 did not reduce the cytotoxic effect of FGF-2-loaded ABNs. The growth-inhibitory and cytotoxic effects of nanoparticle-mediated intracellular delivery of FGF-2 may be broadly applicable to cancer cells that derive from the lung, and perhaps from other tissues/organs. Based on the encouraging data with a lung cancer cell line presented here, future work may explore the influence or activity of ligand receptors outside and inside cancer cells and the intracellular trafficking of ABNs and the release of FGF-2.

## Supplementary Materials

The following are available online at https://www.mdpi.com/2079-4991/10/4/612/s1, Figure S1: Expression and purification of recombinant human FGF-2, Figure S2: Western blot gel images of whole cell lysate from HBE1 and A549 cells with and without FGF-2-loaded ABN treatment, Figure S3: Control cell viability with exogenous FGF-2 and after treatment with FGF-2-loaded ABNs.

## References

[1] Chen Z., Fillmore C.M., Hammerman P.S., Kim C.F., Wong K.-K. (2014). Non-small-cell lung cancers: A heterogeneous set of diseases. Nat. Rev. Cancer.

[2] Jemal A., Bray F., Center M.M., Ferlay J., Ward E., Forman D. (2011). Global cancer statistics. CA Cancer J. Clin..

[3] Siegel R.L., Miller K.D., Jemal A. (2015). Cancer statistics, 2015. CA Cancer J. Clin..

[4] Molina J.R., Yang P., Cassivi S.D., Schild S.E., Adjei A.A. (2008). Non-small cell lung cancer: Epidemiology, risk factors, treatment, and survivorship. Mayo Clin. Proc..

[5] Marina N., Gebhardt M., Teot L., Gorlick R. (2004). Biology and therapeutic advances for pediatric osteosarcoma. Oncologist.

[6] Manegold C. (2001). Chemotherapy for advanced non-small cell lung cancer: Standards. Lung Cancer.

[7] Lee W.L., Guo W.M., Ho V.H.B., Saha A., Chong H.C., Tan N.S., Tan E.Y., Loo S.C.J. (2015). Delivery of doxorubicin and paclitaxel from double-layered microparticles: The effects of layer thickness and dual-drug vs. single-drug loading. Acta Biomater..

[8] Wong H.L., Bendayan R., Rauth A.M., Li Y., Wu X.Y. (2007). Chemotherapy with anticancer drugs encapsulated in solid lipid nanoparticles. Adv. Drug Deliver. Rev..

[9] Wagner E.R., Luther G., Zhu G., Luo Q., Shi Q., Kim S.H., Gao J.-L., Huang E., Gao Y., Yang K. (2011). Defective osteogenic differentiation in the development of osteosarcoma. Sarcoma.

[10] Turner N., Grose R. (2010). Fibroblast growth factor signalling: From development to cancer. Nat. Rev. Cancer.

[11] Brooks A.N., Kilgour E., Smith P.D. (2012). Molecular pathways: Fibroblast growth factor signaling: A new therapeutic opportunity in cancer. Clin. Cancer Res..

[12] Dienstmann R., Rodon J., Prat A., Perez-Garcia J., Adamo B., Felip E., Cortes J., Iafrate A.J., Nuciforo P., Tabernero J. (2014). Genomic aberrations in the FGFR pathway: Opportunities for targeted therapies in solid tumors. Ann. Oncol..

[13] Dieci M.V., Arnedos M., Andre F., Soria J.C. (2013). Fibroblast Growth Factor Receptor Inhibitors as a Cancer Treatment: From a Biologic Rationale to Medical Perspectives. Cancer Discov..

[14] Korc M., Friesel R.E. (2009). The role of fibroblast growth factors in tumor growth. Curr. Cancer Drug Targets.

[15] Touat M., Ileana E., Postel-Vinay S., Andre F., Soria J.C. (2015). Targeting FGFR Signaling in Cancer. Clin. Cancer Res..

[16] Shindo Y., Iwamoto K., Mouri K., Hibino K., Tomita M., Kosako H., Sako Y., Takahashi K. (2016). Conversion of graded phosphorylation into switch-like nuclear translocation via autoregulatory mechanisms in ERK signaling. Nat. Commun..

[17] Kamata T., Hattori Y., Hamada H., Kizaki M., Terada M., Ikeda Y. (2002). Keratinocyte growth factor regulates proliferation and differentiation of hematopoietic cells expressing the receptor gene K-sam. Exp. Hematol..

[18] Caunt C.J., McArdle C.A. (2012). ERK phosphorylation and nuclear accumulation: Insights from single-cell imaging. Biochem. Soc. Trans..

[19] Szybowska P., Kostas M., Wesche J., Wiedlocha A., Haugsten E.M. (2019). Cancer Mutations in FGFR2 Prevent a Negative Feedback Loop Mediated by the ERK1/2 Pathway. Cells.

[20] Presta M., Chiodelli P., Giacomini A., Rusnati M., Ronca R. (2017). Fibroblast growth factors (FGFs) in cancer: FGF traps as a new therapeutic approach. Pharmacol. Ther..

[21] Pardo O.E., Latigo J., Jeffery R.E., Nye E., Poulsom R., Spencer-Dene B., Lemoine N.R., Stamp G.W., Aboagye E.O., Seckl M.J. (2009). The fibroblast growth factor receptor inhibitor PD173074 blocks small cell lung cancer growth in vitro and in vivo. Cancer Res..

[22] Pattarozzi A., Carra E., Favoni R.E., Wurth R., Marubbi D., Filiberti R.A., Mutti L., Florio T., Barbieri F., Daga A. (2017). The inhibition of FGF receptor 1 activity mediates sorafenib antiproliferative effects in human malignant pleural mesothelioma tumor-initiating cells. Stem Cell Res. Ther..

[23] Gaubert F., Escaffit F., Bertrand C., Korc M., Pradayrol L., Clemente F., Estival A. (2001). Expression of the high molecular weight fibroblast growth factor-2 isoform of 210 amino acids is associated with modulation of protein kinases C delta and epsilon and ERK activation. J. Biol. Chem..

[24] Sorensen V., Nilsen T., Wiedlocha A. (2006). Functional diversity of FGF-2 isoforms by intracellular sorting. Bioessays.

[25] Lemiere S., Azar R., Belloc F., Gursel D., Pyronnet S., Bikfalvi A., Auguste P. (2008). Overexpression of high molecular weight FGF-2 forms inhibits glioma growth by acting on cell-cycle progression and protein translation. Exp. Cell Res..

[26] Yoon S., Seger R. (2006). The extracellular signal-regulated kinase: Multiple substrates regulate diverse cellular functions. Growth Factors.

[27] Catalgol B., Batirel S., Taga Y., Ozer N.K. (2012). Resveratrol: French Paradox Revisited. Front. Pharmacol..

[28] Lee J.H., Kim K.T. (2007). Regulation of cyclin-dependent kinase 5 and p53 by ERK1/2 pathway in the DNA damage-induced neuronal death. J. Cell. Physiol..

[29] Cagnol S., Chambard J.C. (2010). ERK and cell death: Mechanisms of ERK-induced cell death--apoptosis, autophagy and senescence. FEBS J..

[30] Liu J., Mao W., Ding B., Liang C.S. (2008). ERKs/p53 signal transduction pathway is involved in doxorubicin-induced apoptosis in H9c2 cells and cardiomyocytes. Am. J. Physiol. Heart C.

[31] Drosten M., Sum E.Y., Lechuga C.G., Simon-Carrasco L., Jacob H.K., Garcia-Medina R., Huang S., Beijersbergen R.L., Bernards R., Barbacid M. (2014). Loss of p53 induces cell proliferation via Ras-independent activation of the Raf/Mek/Erk signaling pathway. Proc. Natl. Acad. Sci. USA.

[32] Maloof P., Wang Q., Wang H., Stein D., Denny T.N., Yahalom J., Fenig E., Wieder R. (1999). Overexpression of basic fibroblast growth factor (FGF-2) downregulates Bcl-2 and promotes apoptosis in MCF-7 human breast cancer cells. Breast Cancer Res. Treat..

[33] Berger W., Setinek U., Mohr T., Kindas-Mugge I., Vetterlein M., Dekan G., Eckersberger F., Caldas C., Micksche M. (1999). Evidence for a role of FGF-2 and FGF receptors in the proliferation of non-small cell lung cancer cells. Int. J. Cancer.

[34] Sinha V.R., Trehan A. (2003). Biodegradable microspheres for protein delivery. J. Control. Release.

[35] Wu Y.Q., MacKay J.A., McDaniel J.R., Chilkoti A., Clark R.L. (2009). Fabrication of Elastin-Like polypeptide Nanoparticles for Drug Delivery by Electrospraying. Biomacromolecules.

[36] Lemoine D., Wauters F., Bouchend’homme S., Preat V. (1998). Preparation and characterization of alginate microspheres containing a model antigen. Int. J. Pharm..

[37] Pawar S.N., Edgar K.J. (2012). Alginate derivatization: A review of chemistry, properties and applications. Biomaterials.

[38] Lee K.Y., Mooney D.J. (2012). Alginate: Properties and biomedical applications. Prog. Polym. Sci..

[39] Tonnesen H.H., Karlsen J. (2002). Alginate in drug delivery systems. Drug Develop. Indust. Pharm..

[40] Cheng J., Pun S.H. (2015). Polymeric biomaterials for cancer nanotechnology. Biomater. Sci..

[41] Fenn S.L., Miao T., Scherrer R.M., Oldinski R.A. (2016). Dual-Cross-Linked Methacrylated Alginate Sub-Microspheres for Intracellular Chemotherapeutic Delivery. ACS Appl. Mater. Interfaces.

[42] Sambrook J., Russell D.W. (2001). Molecular Cloning: A Laboratory Manual.

[43] Miao T., Rao K.S., Spees J.L., Oldinski R.A. (2014). Osteogenic differentiation of human mesenchymal stem cells through alginate-graft-poly(ethylene glycol) microsphere-mediated intracellular growth factor delivery. J. Control. Release.

[44] Gåserød O., Sannes A., Skjåk-Bræk G. (1999). Microcapsules of alginate–chitosan. II. A study of capsule stability and permeability. Biomaterials.

[45] Yankaskas J.R., Haizlip J.E., Conrad M., Koval D., Lazarowski E., Paradiso A.M., Rinehart C.A., Sarkadi B., Schlegel R., Boucher R.C. (1993). Papilloma virus immortalized tracheal epithelial cells retain a well-differentiated phenotype. Am. J. Physiol..

[46] Shimoda A., Sawada S.I., Akiyoshi K. (2011). Cell Specific Peptide-Conjugated Polysaccharide Nanogels for Protein Delivery. Macromol. Biosci..

[47] Lee R.J., Wang S., Low P.S. (1996). Measurement of endosome pH following folate receptor-mediated endocytosis. Biochim. Biophys. Acta.

[48] Huang R., Ke W., Han L., Liu Y., Shao K., Ye L., Lou J., Jiang C., Pei Y. (2009). Brain-targeting mechanisms of lactoferrin-modified DNA-loaded nanoparticles. J. Cerebral Blood Flow Metab..

[49] Preta G., Cronin J.G., Sheldon I.M. (2015). Dynasore—Not just a dynamin inhibitor. Cell Commun. Signal..

[50] Kirchhausen T., Macia E., Pelish H.E. (2008). Use of dynasore, the small molecule inhibitor of dynamin, in the regulation of endocytosis. Methods Enzymol..

[51] Yin S., Huai J., Chen X., Yang Y., Zhang X., Gan Y., Wang G., Gu X., Li J. (2015). Intracellular delivery and antitumor effects of a redox-responsive polymeric paclitaxel conjugate based on hyaluronic acid. Acta Biomater..

[52] Baravalle G., Schober D., Huber M., Bayer N., Murphy R.F., Fuchs R. (2005). Transferrin recycling and dextran transport to lysosomes is differentially affected by bafilomycin, nocodazole, and low temperature. Cell Tissue Res..

[53] Mundy D.I., Li W.P., Luby-Phelps K., Anderson R.G.W. (2012). Caveolin targeting to late endosome/lysosomal membranes is induced by perturbations of lysosomal pH and cholesterol content. Mol. Biol. Cell.

[54] Hou X., Lewis K.T., Wu Q., Wang S., Chen X., Flack A., Mao G., Taatjes D.J., Sun F., Jena B.P. (2014). Proteome of the porosome complex in human airway epithelia: Interaction with the cystic fibrosis transmembrane conductance regulator (CFTR). J. Proteom..

[55] Wang S., Lee J.S., Bishop N., Jeremic A., Cho W.J., Chen X., Mao G., Taatjes D.J., Jena B.P. (2012). 3D organization and function of the cell: Golgi budding and vesicle biogenesis to docking at the porosome complex. Histochem. Cell Biol..

[56] Sham D., Wesley U.V., Hristova M., van der Vliet A. (2013). ATP-mediated transactivation of the epidermal growth factor receptor in airway epithelial cells involves DUOX1-dependent oxidation of Src and ADAM17. PLoS ONE.

[57] Nam H.Y., Kwon S.M., Chung H., Lee S.-Y., Kwon S.-H., Jeon H., Kim Y., Park J.H., Kim J., Her S. (2009). Cellular uptake mechanism and intracellular fate of hydrophobically modified glycol chitosan nanoparticles. J. Control. Release.

[58] Kou L., Sun J., Zhai Y., He Z. (2013). The endocytosis and intracellular fate of nanomedicines: Implication for rational design. Asian J. Pharm. Sci..

[59] Rejman J., Oberle V., Zuhorn I.S., Hoekstra D. (2004). Size-dependent internalization of particles via the pathways of clathrin-and caveolae-mediated endocytosis. Biochem. J..

[60] Boussif O., Lezoualc’h F., Zanta M.A., Mergny M.D., Scherman D., Demeneix B., Behr J.P. (1995). A versatile vector for gene and oligonucleotide transfer into cells in culture and in vivo: Polyethylenimine. Proc. Natl. Acad. Sci. USA.

[61] Zhang C., Shi G., Zhang J., Song H., Niu J., Shi S., Huang P., Wang Y., Wang W., Li C. (2017). Targeted antigen delivery to dendritic cell via functionalized alginate nanoparticles for cancer immunotherapy. J. Control. Release.

[62] Ma X., Dang X., Claus P., Hirst C., Fandrich R.R., Jin Y., Grothe C., Kirshenbaum L.A., Cattini P.A., Kardami E. (2007). Chromatin compaction and cell death by high molecular weight FGF-2 depend on its nuclear localization, intracrine ERK activation, and engagement of mitochondria. J. Cell. Physiol..

[63] Choi J., Ko M.K., Kay E.P. (2000). Subcellular localization of the expressed 18 kDa FGF-2 isoform in corneal endothelial cells. Mol. Vis..

[64] Sheng Z., Lewis J.A., Chirico W.J. (2004). Nuclear and nucleolar localization of 18-kDa fibroblast growth factor-2 is controlled by C-terminal signals. J. Biol. Chem..

[65] Claus P., Doring F., Gringel S., Muller-Ostermeyer F., Fuhlrott J., Kraft T., Grothe C. (2003). Differential intranuclear localization of fibroblast growth factor-2 isoforms and specific interaction with the survival of motoneuron protein. J. Biol. Chem..

