# Supplementary material for: Internalized FGF-2-Loaded Nanoparticles Increase Nuclear ERK1/2 Content and Result in Lung Cancer Cell Death
Source: Nanomaterials (Basel). 2020 Mar 27;10(4):612. doi: 10.3390/nano10040612 (PMC7221911; doi:10.3390/nano10040612)
Supplement: Supplementary file 1 [file nanomaterials-10-00612-s001.pdf]

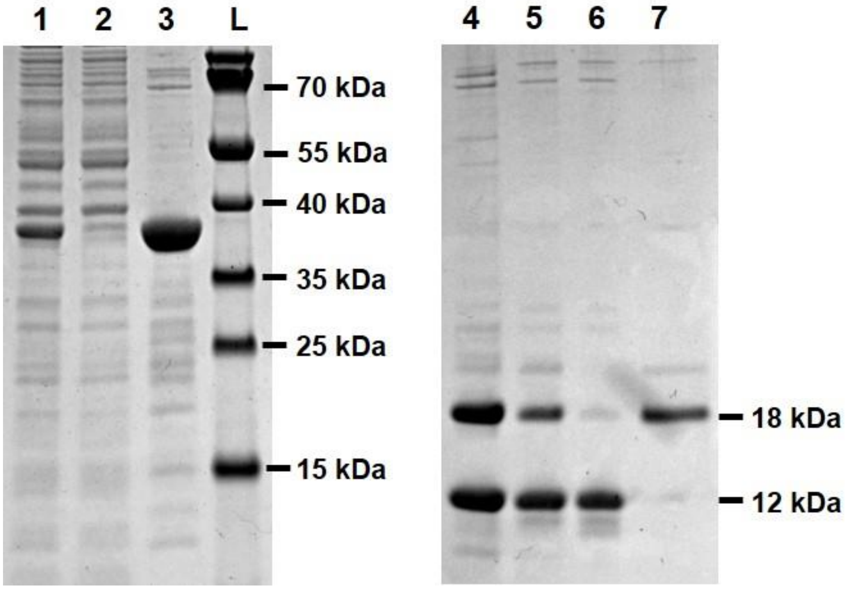

Figure S1. Expression and purification of recombinant human FGF-2 (18 kDa isoform) from *E. coli.* Lane 1: Supernatant of *E. coli* crude extract applied onto Ni-NTA column. Lane 2: Flow-through fraction from Ni-NTA column. Lane 3: Eluate from Ni-NTA column. Lane 4: Thrombin cleavage of the thioredoxin-FGF-2 fusion protein eluted from Ni-NTA column (FGF-2, 18 kDa; thioredoxin, 12 kDa). Lane 5: Loading onto heparin-sepharose column. Lane 6: Flow-through fraction from heparin-sepharose (thioredoxin). Lane 7: Eluate from heparin-sepharose (FGF-2).

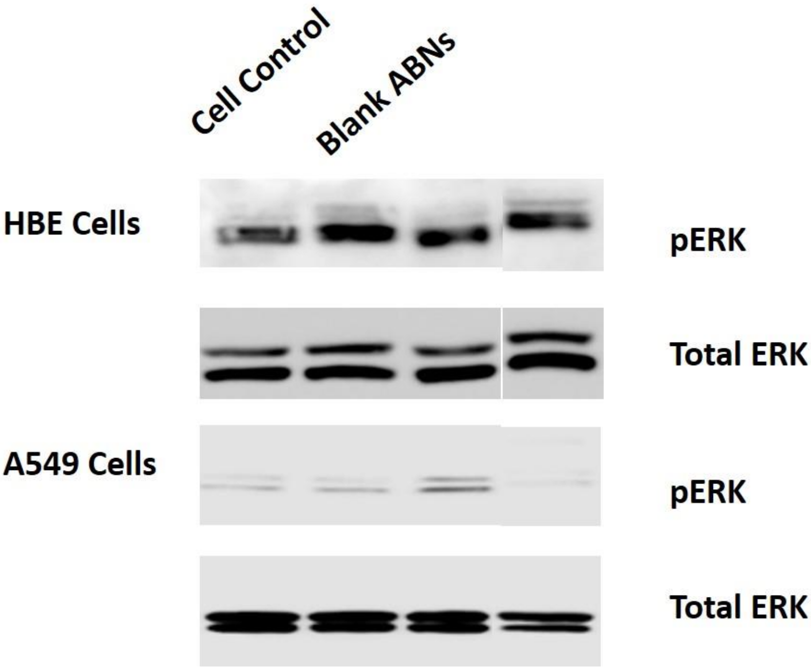

Figure S2. Western blot gel images of whole cell lysate from HBE1s and A549s with and without FGF-2-loaded ABN treatment.

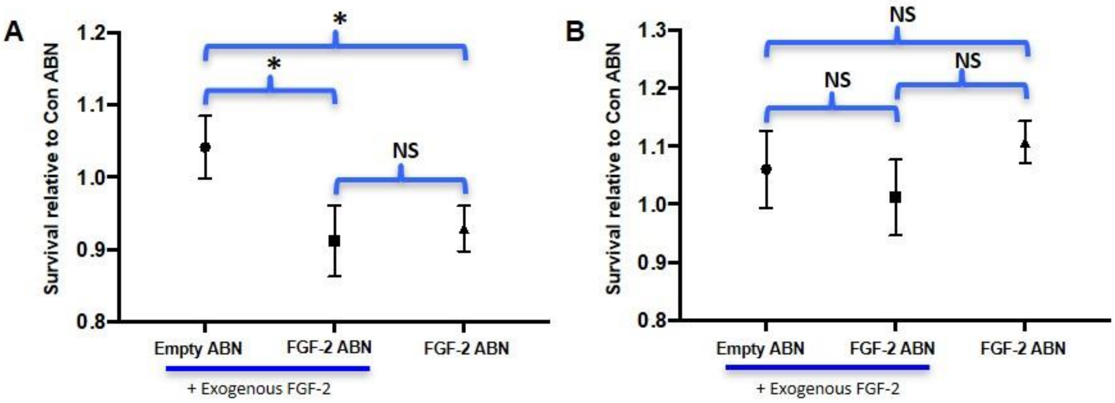

Figure S3. Treatment with FGF-2-loaded ABNs reduces the number of (**A**) A549s, but not (**B**) HBE1 cells, even in the presence of free FGF-2. Each cell type was grown in their respective mediums and then transferred to a mixed A549/HBE1 medium (1:1) for 48 h. Control ABNs (empty) or FGF-2-loaded ABNs were suspended in mixed medium with exogenous FGF-2, and added at 100 μg/mL to different wells. FGF-2-loaded ABNs were also examined with no additional FGF-2 added to the medium. After 24 h, cell number was determined using CyQuant Direct Cell Proliferation Assay (n = 3-4). * $P < 0.05$; NS = Not Significant. A.U. = Arbitrary Units.
